# Integration of a Virtual Dispensing Simulator “MyDispense” in an Experiential Education Program to Prepare Students for Community Introductory Pharmacy Practice Experience

**DOI:** 10.3390/pharmacy9010048

**Published:** 2021-02-27

**Authors:** Ashley E. Johnson, Jillian Barrack, Jill M. Fitzgerald, Diana M. Sobieraj, Lisa M. Holle

**Affiliations:** 1 Dartmouth-Hitchcock Medical Center, Lebanon, NH 03756, USA; ashley.e.johnson@hitchcock.org; 2 St. Francis Hospital and Medical Center, Hartford, CT 06105, USA; jillian.barrack@trinityhealthofne.org; 3 University of Connecticut School of Pharmacy, Department of Pharmacy Practice, Storrs, CT 06269, USA; jill.fitzgerald@uconn.edu (J.M.F.); diana.sobieraj@uconn.edu (D.M.S.)

**Keywords:** MyDispense, simulation, introductory pharmacy, skills

## Abstract

Background: Technology is increasingly used to enhance pharmacy education. We sought to evaluate student learning and preparedness for community introductory pharmacy practice experiences (IPPEs) after implementation of “MyDispense” into experiential education. Methods: Both first-year pharmacy students and assigned community IPPE preceptors were eligible. Students were stratified based on previous community pharmacy experience (< or ≥ 50 h), then randomized to complete MyDispense exercises before IPPE (group A) or after 24–32 h of IPPE (group B). We evaluated preceptors’ assessment of student readiness using a 6-item Likert scale survey and students’ readiness and opinion of MyDispense using an anonymous 9-item survey. Descriptive statistics were used to characterize data. The Mann–Whitney U test was used to compare groups and a *p*-value < 0.05 was considered statistically significant. Results: Of 177 eligible students, 155 were randomized and 56 completed study. Group A included 32 students; 56.3% had prior community practice experience. Group B included 24 students; 50% had prior community practice experience. Forty-eight preceptors were enrolled. Students who completed exercises before rotation received higher preceptor scores for patient counseling of self-care and of medications (*p* < 0.05 for both). Students self-assessed their counseling skills lower than all other skills; 30.4% and 42.9% of students felt mostly or always prepared to counsel for self-care and medications, respectively. Students found MyDispense straightforward, realistic, and appreciated the ability to practice in a safe, electronic, community pharmacy, patient-care environment. Conclusion: Simulation-based software, such as MyDispense, can enhance learner understanding of the prescription fill and counseling process in a community pharmacy practice setting.

## 1. Introduction

As technology becomes more ubiquitous throughout education, institutions of professional healthcare learning are incorporating various tools for students to utilize [1,2,3,4,5,6,7,8,9,10,11,12,13,14,15,16]. A 2011 survey of 89 pharmacy schools in the United States reported the use of some forms of technology ranging from lecture capture and presentation software to videoconferencing and social media. In this study, schools reported 100% use of technology for course management purposes, 79.8% use of technology for electronic testing, and 58.4% use of technology for experiential education programs [17]. Similarly, a 2015 review of the available literature also detailed a large variety of technology being used in schools of pharmacy for improving patient care [18]. Schools of pharmacy are embracing technology, and their students are as well. A survey in 2016 of 431 pharmacy students found that students preferred a blended approach to learning and not an online-only or in lecture-only approach [19]. These studies represent the desire of pharmacy schools and students to utilize technology in traditional and nontraditional educational settings. However, a 2014 review of 17 studies of the available literature determined that little evidence exists to support the use of e-learning in improving skills for professional practice [20]. The review also questioned whether e-learning is effective at increasing knowledge long-term. Currently, limited literature is available on the evaluation of simulation technology use in pharmacy education [17,18].

The purpose of this study was to assess the effectiveness of a simulation technology on improving the clinical dispensing skills of pharmacy students. First-year pharmacy students (P1) at the University of Connecticut (UConn) School of Pharmacy were introduced to an online virtual dispensing software entitled MyDispense in experiential education classes. MyDispense was developed by the Faculty of Pharmacy and Pharmaceutical Sciences, Monash University [21,22]. The software places the student in the role of a community pharmacist, where the student is charged with interacting with virtual patients in various exercises simulating dispensing medications, counseling patients, and collaborating with virtual providers to optimize patient care. Exercises can be programmed to offer the student different types of challenges from a patient presenting a forged prescription to a patient uncertain of how to best manage her diabetes. By practicing in a safe environment, students can gain confidence in their dispensing and clinical skills while receiving immediate feedback on their performance [23].

Beginning in 2013, Monash, in conjunction with faculty from UConn, the University of California, San Francisco (UCSF), and the St. Louis College of Pharmacy (STLCOP), adapted the MyDispense program for use within the United States [24]. This process included updating the program to reflect current US laws and practices [25]. One example is the use of amber vials and counting loose tablets to dispense the prescribed amount. In Australia, medications are not dispensed as loose tablets, but in the manufactured packaged unit. As a result, a strong emphasis is placed on the correct placement of the prescription label on the bottle. This practice is not as common in the US where pharmacists and technicians count out the prescribed amount of medication and place the loose tablets within a vial. As a result, a counting tray was added to the MyDispense process to ensure student skill development. The medications also needed to be adapted to include names and images of US prescription stock bottles, which also differed from their Australian counterparts.

At UConn, the software was selected for initial integration into the experiential education program. As MyDispense currently only simulates a community pharmacy, the program was incorporated into the Introductory Pharmacy Practice Experience (IPPE) Community Pharmacy course for first-year pharmacy students. Within this course, students were assigned to individual preceptors and practice sites and had to complete the 100- to 120-h rotation between the end of November and beginning of April of their first year. For some of these students, this was their first experience working in a community pharmacy. This placed them at the disadvantage of being relegated to observation. In turn, these students then lost the opportunity to maximize their hours to fully develop their skillset.

Through the incorporation of MyDispense, students gained an opportunity to familiarize themselves with a community pharmacy prior to engaging in their rotations. With such advanced preparation, preceptors gain more knowledgeable and capable students, allowing them to delve further into practical and clinical skills.

The purpose of this prospective study was to evaluate the addition of a virtual dispensing software in an experiential education program and the associated outcomes on student learning and preparedness for IPPE rotations.

## 2. Materials and Methods

This two-year study was designed as a prospective, randomized trial with parallel study groups. The UConn Institutional Review Board approved this study. All P1 students in the School of Pharmacy and enrolled in the course PHRX 3020 IPPE I: Community Pharmacy during the 2015–2016 and 2016–2017 academic years were eligible for participating in the study. Additionally, the community pharmacy preceptors assigned to each student were eligible for participating in the study. Students and preceptors were blinded to the others’ participation in the study to decrease the risk of grading bias. Half of the investigators remained blinded to contributing to data analysis, while the remainder of the team was unblinded to ensure the study timeline was kept.

Students were presented with the opportunity to take part in the study by faculty and student investigators, and the MyDispense software was introduced. After providing consent, students completed a brief demographic survey and were stratified based on their previous community practice experience using 50 h or more of work experience as a threshold. We then randomized students to complete the MyDispense assignments before IPPE (group A) or after the first 24–32 h of their IPPE experience (group B).

After randomization, the students received an email from an unblinded researcher that explained whether the student needed to complete their MyDispense exercises before the first day of the IPPE experience or after the rotation began. Once all students were notified, students in group A were granted access to the MyDispense exercises. These students were required to complete all exercises by the start of their rotation schedule. Students in group B were granted access to the assigned exercises after their preceptors completed the student readiness survey when 24–32 h of the IPPE hours were completed. If the preceptors of any of the students in group B declined to participate, students were granted access to the assignment after beginning their rotation. However, these student data were not included in the analysis.

A total of 40 MyDispense exercises were assigned to each student. All 40 exercises had to be completed regardless of group assignment. Students who completed all 40 exercises received a maximum allotment of 10 h towards their required IPPE experiential hours. Students who did not complete all 40 exercises, or did not complete the exercises in the required timeframe, did not receive 10 IPPE hours and were not included in the data analysis. Students who elected not to participate in the study were granted access to the MyDispense exercises for personal benefit, but did not receive credit toward their required IPPE experiential hours.

The assigned exercises varied in pharmacy subject area and complexity. In most instances, the student was presented with a virtual patient who was dropping off a prescription. The student had to first determine if the prescription was appropriate and legal to fill, and then had to accurately fill the prescription with the proper label, medication and quantity, and auxiliary labels. The student was able to contact the virtual prescriber with questions if needed in specific exercises. The student was expected to ask relevant questions of the patient and effectively respond to the patient’s questions, as well as counsel the patient on their medication before dispensing. Other exercises included making over-the-counter product recommendations, determining safe controlled drug dispensing practices, and medication identification. All assigned exercises were created and reviewed by members of the research team prior to release to students.

Preceptors were invited to take place in the study after being assigned to a student pharmacist. An unblinded investigator sent out an informational sheet on the study to preceptors via the email function on the School of Pharmacy’s externship system, RxPreceptor (West Warwick, RI, USA). Preceptors were invited to respond if interested, and if they expressed a willingness to participate, they were assigned a number designation for blinding purposes by an unblinded researcher. Preceptors were then sent a link to complete a student readiness survey for their student via RxPreceptor. The preceptors were instructed to complete the student readiness survey after their assigned student had completed 24–32 IPPE hours. This time period was decided upon to gain perspective on the preceptor’s initial thoughts of the student’s level of preparedness to effectively function in a community pharmacy. For students in group B, their MyDispense exercises were released to them after their preceptor had completed the survey, as described above. The student readiness survey contained six questions and evaluated student performance on a six-point Likert scale (Table 1) with 1 meaning “unacceptable” and 6 meaning “excellent”. The survey was administered through Qualtrics online survey software (Provo, Utah).

Following exercises completion, students were then asked to complete a survey evaluating their performance during their rotation as well as the usefulness of the MyDispense program. The survey consisted of nine questions, each of which the students evaluated using a five-point Likert scale, with 1 meaning “not at all” and 5 “all of the time” (Table 2). The survey link was provided by email and administered through Qualtrics online survey software (Provo, UT, USA). Each student was given until April of their academic year to complete the survey.

Demographics of students were descriptively reported using frequencies. Results of each survey question were summarized using medians with an accompanying interquartile range. Our primary analysis was to compare preceptor survey responses from students of group A versus group B. Group A and group B student data without matching preceptor data were not included in the analysis. Student survey responses were summarized in aggregate due to the anonymous format of the student surveys. Finally, we compared response from preceptors with those of students. Groups were compared with the Mann–Whitney U test. We determined statistical significance with a *p*-value less than 0.05.

## 3. Results

Of the 177 first-year pharmacy students eligible for study participation, 155 students enrolled in the study and 56 (36%) students completed the survey (32 in group A and 24 in group B) (Table 3). We received surveys from 48 preceptors. More than half of participating students had pharmacy work experience (64.3%). Most of the experience was in community pharmacies (53.6%), followed by institutional pharmacies (8.9%). Previous community pharmacy experience was similar between groups, with 18 (56.3%) and 12 (50%) students having such experience in groups A and B, respectively. Overall, chain community pharmacy was more common than independent pharmacy experience. Demographic information for preceptors was not collected.

Preceptor evaluations of student performance for students who completed MyDispense exercises before their community IPPE (group A) were at or above the median score for students who completed them after the preceptor assessment (group B) (Table 4). Preceptor scores for patient counseling were significantly higher for students who completed MyDispense exercises before their community IPPEs (*p* < 0.05), but not for other community pharmacy activities. We performed a subgroup analysis based on students’ previous community pharmacy experience. In the group without prior community practice experience (less than 50 total hours), completing MyDispense activities before IPPE led to higher preceptor scores on self-care counseling skills (median 4 vs. 3, *p* = 0.05) and medication counseling (median 4 vs. 3.5, *p* = 0.04) compared to students completing the activities later. No significant difference was found in any preceptor assessments within the subgroup of students with prior community practice experience.

Student assessment of their community pharmacy skills following MyDispense learning varied (Table 5). The lowest median scores were associated with the student’s ability to counsel patients on both medications and self-care, as well as the student’s ability to provide basic medication counseling at an appropriate level to the patient and caregiver. The highest scores were found in being able to correctly interpret or translate prescription abbreviations (87.5% scored 4 or 5 on Likert score) and select the correct drug product, dose, and dosage form (92.9% scored 4 or 5 on Likert score) (see Table 5). When asked to evaluate their perception of MyDispense, most students felt it was straightforward to learn (51.8%), was more realistic than solving cases on paper (60.7%), and provided an opportunity to develop skills without associated patient harm (85.7%).

## 4. Discussion

This is the first study evaluating the use of MyDispense in preparing first-year pharmacy students for IPPE rotations in community pharmacy. This study specifically evaluated preceptor perception of student preparedness based on the timing of program exposure, while also stratifying students based on previous community pharmacy experience. Additionally, student perceptions of self and of the program were evaluated. Students generally rated themselves highly across all survey topics with median Likert scores of 3 or higher. In regards to counseling patients on either medications or preventative care, students felt less confident. This may be attributed to the simulation aspect of MyDispense, which cannot fully mimic the intricacies of human behavior or interactions. Additionally, feedback on counseling was given with all ideal counseling points being made, which may not be synchronous with all patient scenarios encountered in practice. Furthermore, the students involved in the study were in their first professional year of pharmacy school. The level of clinical knowledge they had accumulated at this point in their career was not extensive even after completing the 40 MyDispense exercises, so this may be another reason for the lack of confidence in counseling patients.

Although most students found the program both easy to learn and more realistic than paper-based patient cases (61%); about one-half of students reported that about 50% of the time or more, MyDispense was not straightforward to use. In another study evaluating MyDispense within a course that practices outpatient pharmacy skills, 84% of students overall reported they found MyDispense useful in preparing them for outpatient pharmacy activities, regardless of previous pharmacy experience (40% of students had no community pharmacy experience) [9]. Therefore, our results may indicate that the orientation to MyDispense may not have been adequate and perhaps more time could be spent introducing the program and allowing time for students to ask questions about usage after the initial introduction.

A similar study published by the University of Wisconsin-Madison School of Pharmacy [26] evaluated students’ self-reported confidence and engagement in the use of a paper-based versus a simulated patient case. Similar to the UConn students, the University of Wisconsin students agreed that the simulated patient case resulted in increased enjoyment (71%), realism (75%), and learning of new content (60%) as compared to a paper-based patient case. These higher satisfaction and engagement ratings may also be attributed to the fact that the University of Wisconsin students were in their third year of pharmacy school, allowing for greater ease of identifying applicability to practice. Another study published by Taipei Medical University [27] evaluated pharmacy students’ self-reported confidence in the use of “modified information delivery” via a simulation patient. The researchers designed stations for the students to go through for each patient: (1) History and lab data collection; (2) prescription review; (3) calling the physician to discuss potential problems; and (4) patient education (counseling). Similar to the UConn students, the Taipei Medical University Students agreed that the simulated patient case was relevant to clinical practice, improved their clinical skills, and was helpful for future work. A total of 86% of the students at Taipei Medical University expressed they would like to see the practice used again in their education. These high satisfaction ratings may also be attributed to the fact that the Taipei Medical University students were in their final year of pharmacy school and about to graduate, allowing for greater ease of identifying applicability to practice. Together with previously published evaluations of teaching technologies [24,25,26,27], these studies corroborate the idea that students prefer simulated patient cases to paper-based.

In contrast to student self-evaluations, preceptors rated students higher on counseling. Students who completed MyDispense exercises before their community IPPEs (group A) were deemed better prepared to provide medication counseling compared to students who did not complete MyDispense exercises (group B). Preceptors rated students in both groups equally poorly (median Likert score 3) in regards to processing of prescriptions, with no difference seen between those with or without previous community pharmacy exposure. This may be attributed to differences in practice between the program and individual store or company policy, as well as the difference in performing a simulated versus a physical task.

Statistical significance was not achieved, although scores were numerically higher in gathering and organizing patient-specific information for those students completing MyDispense activities before their community IPPE and those completing after preceptor rating their community pharmacy readiness. This pattern of non-significant but numerically higher results between the 2 groups continued for the student’s ability to interpret prescription abbreviations and to select the appropriate drug product, dose, and dosage form.

Overall, MyDispense did not statistically prove to be an effective teaching tool in preparing students for all community pharmacy IPPE activities rotations. However, the limitation in preceptor and student response may contribute to a lack of significance in these results. The trend in numerically higher scores for students completing MyDispense activities before their community pharmacy IPPE suggests the possibility that MyDispense can help students become more prepared for rotations.

Another possible cause for the non-significant results in terms of improvements noted by preceptors is that the exercises included were perhaps not the ones most needed to improve IPPE skills. For example, exercises regarding over-the-counter (OTC) medications were not included. OTC recommendations and counseling are important facets of the skillset of a community pharmacist, so students who had practiced these skills in the MyDispense exercises may have scored higher.

This study was limited by the sample population. Despite most students enrolling in the study, we received a low survey response rate. Given that students had a five-month window in which to complete their rotation, full adherence to study protocol was difficult to assess. Student survey responses were both voluntary in nature and anonymous. This limited both number of responses as well as the true extent of analysis that could be performed. Preceptor participation in particular was very low, which likely reflects the difficulty in communication experienced. Email was the sole method of communication to preceptors, which coupled with limited follow-up and the voluntary nature of participation, likely diminished enrollment. Preceptor survey results were also complicated by the disproportionate numbers of enrolled preceptors and corresponding student group enrollment. Similar to preceptor participation, student participation was strictly voluntary. Those students who elected not to participate were still granted access to the MyDispense study exercises for additional skill development as desired. No information on student preparedness was collected from preceptors whose assigned students chose not to participate.

The positive results of this study, along with student feedback, prompted the incorporation of MyDispense exercises as an addition to the curriculum at the UConn of Pharmacy. Exercises have been edited and further developed to include subjects previously excluded, such as OTC recommendations, for first-year students. Additionally, MyDispense exercises were introduced to students in all professional years to aid in learning activities of various subjects such as practicing dispensing in skills labs throughout the curriculum, improving understanding of pharmacy law, and practicing law concepts, counseling, and prescription review process within a pain management course. More recently, both within our own institution and throughout the world, MyDispense has been used to supplement experiential learning when remote/online teaching was required during the COVID-19 pandemic, indicating another potential use for MyDispense [28,29].

## 5. Conclusions

Simulation-based learning, such as MyDispense, offers an effective teaching tool in preparing students for rotational experiences in community pharmacy. Although this study did not demonstrate that preceptors view students as more prepared for their experimental rotations after using the software, the program has further benefits. The program is particularly useful in fostering students’ confidence in their developing skillset, and may be utilized throughout their professional years to help them practice in whichever area being studied at the time. Further study is needed to better quantify the exact benefits of the MyDispense program.

## Figures and Tables

**Table 1:** Preceptor survey questions evaluating student readiness for community introductory pharmacy practice experience. ^1^

**Survey Questions:** The student was able to gather and organize accurate and comprehensive patient specific information.
The student can accurately evaluate and process a new prescription or prescription refill, in a time-sensitive manner.
The student was able to correctly interpret/translate prescription abbreviations.
The student was able to select the correct drug product, dose, and dosage form.
The student was able to counsel the patients on proper self-care and preventative care to an appropriate level.
The student was able to provide basic medication counseling to a patient or caregiver receiving a medication to an appropriate level.

^1^ 6-point Likert scale responses: 1 = unacceptable; 2 = needs significant development; 3 = needs development; 4 = fair; 5 = good; 6 = excellent.

**Table 2:** Survey questions evaluating student performance during community introductory pharmacy practice experience. ^1^

**Survey Questions:** I am able to gather and organize accurate and comprehensive patient specific information.
I am able to accurately evaluate and process a new prescription or prescription refill, in a time-sensitive manner.
I am able to correctly interpret/translate prescription abbreviations.
I am able to select the correct drug product, dose, and dosage form.
I am able to counsel the patients on proper self-care and preventative care to an appropriate level.
I am able to provide basic medication counseling to a patient or caregiver receiving a medication to an appropriate level.
In my experience, MyDispense was straightforward to learn.
In my experience, using the MyDispense program to address patient issues (questioning, dispensing, counseling) was more realistic than addressing similar patient cases on paper.
I appreciated that MyDispense afforded me the opportunity to make some mistakes with dispensing, but knowing that patients would not be harmed by these errors.

^1^ 5-point Likert scale responses: 1 = not at all; 2 = some of the time (1–40% of the time); 3 = about 50% of the time; 4 = most of the time (60–99% of the time); 5 = all of the time (100% of the time).

**Table 3:** Demographic information.

Student Previous Experience, ^1^ *n* (%)	Student Group A (*n* = 32)	Student Group B (*n* = 24)
Community Pharmacy	18 (56.3)	12 (50.0)
Independent	6 (18.8)	1 (4.2)
Chain	12 (37.5)	11 (45.8)
Multiple Sites	0 (0)	2 (8.3)
Institutional	4 (12.5)	1 (4.2)
Other	1 (3.1)	0 (0)
Preceptors enrolled, *n*	22	26

^1^ Previous or current experience.

**Table 4:** Preceptor analysis of student readiness. ^1.^

Survey Question, Median (IQR)	Student Group A (*n* = 32)	Student Group B (*n* = 24)	*p*-Value
The student was able to gather and organize accurate and comprehensive patient specific information.	3 (2 to 5.75)	2 (2 to 5)	0.392
The student can accurately evaluate and process a new prescription or prescription refill, in a time-sensitive manner.	3 (2 to 5.75)	3 (2 to 5)	0.541
The student was able to correctly interpret/translate prescription abbreviations.	4 (2.25 to 6)	3 (2 to 5)	0.258
The student was able to select the correct drug product, dose, and dosage form.	3 (2 to 6)	2 (2 to 5.75)	0.393
The student was able to counsel the patients on proper self-care and preventative care to an appropriate level.	4 (4 to 5)	3 (3 to 4)	0.015
The student was able to provide basic medication counseling to a patient or caregiver receiving a medication to an appropriate level,	4 (3.25 to 5.75)	3.5 (2.25 to 4)	0.027

^1^ 6-point Likert scale responses: 1 = unacceptable; 2 = needs significant development; 3 = needs development; 4 = fair; 5 = good; 6 = excellent.

**Table 5:** Student performance analysis. ^1.^

Survey Question	Student Response Median (IQR) (*n* = 56)	Student Response 4 or 5 on Likert Scale, *n* (%) (*n* = 56)
I am able to gather and organize accurate and comprehensive patient specific information.	4 (3 to 4)	40 (71.7)
I am able to accurately evaluate and process a new prescription or prescription refill, in a time-sensitive manner.	4 (3 to 4)	38 (67.9)
I am able to correctly interpret/translate prescription abbreviations.	4 (4 to 5)	49 (87.5)
I am able to select the correct drug product, dose, and dosage form.	4 (4 to 5)	52 (92.9)
I am able to counsel the patients on proper self-care and preventative care to an appropriate level.	3 (3 to 4)	17 (30.4)
I am able to provide basic medication counseling to a patient or caregiver receiving a medication to an appropriate level.	3 (3 to 4)	24 (42.9)
In my experience, MyDispense was straightforward to learn.	4 (3 to 4)	29 (51.8)
In my experience, using MyDispense program to address patient issues (questioning, dispensing, counseling) was more realistic than addressing similar patient cases on paper).	4 (3 to 4)	34 (60.7)
I appreciated that MyDispense afforded me the opportunity to make some mistakes with dispensing, but knowing that patients would not be harmed by these errors.	5 (4 to 5)	48 (85.7)

^1^ 5-point Likert scale responses: 1 = not at all; 2 = some of the time (1–40% of the time); 3 = about 50% of the time; 4 = most of the time (60–99% of the time); 5 = all of the time (100% of the time).

## Data Availability

The data presented in this study may be available on request from the corresponding author. The data are not publicly available due to ethical and privacy concerns.
